# The Value of Sneezing in the Reduction of Hernia

**Published:** 1877-04-20

**Authors:** Charles Denison

**Affiliations:** Denver, Col.


### THE VALUE OF SNEEZING IN THE REDUCTION OF HERNIA.

By CHARLES DENISON, M. D., Denver, Col.

As I am not aware that the procedure of giving snuff, while an attempt at reduction of hernia by taxis is being made, has been known to the profession, I wish to report two instances which have lately occurred in my practice;

May 30. I was called out at night to see a Chicago gentleman, temporarily sojourning at the Evergreen House in Morrison. I reached him about half-past four next morning, having to go *via* Mt. Vernon, around the "Hog-backs," or outside foot hills of the Rocky Mountains. I found Dr. J. C. Dunham in charge, from whom I heard that Mr. C. had the previous evening caused the hernia while helping a lady to alight from a carriage; that he had had the difficulty for several years, during which time he had worn a truss, and had two or three times suffered from its strangulation, due to his negligence in leaving off his truss. It was oblique, inguinal hernia, and this time the protrusion was sufficient to cause the right side of the scrotum to assume the size of a man's fist. Dr. Dunham had used sufficient taxis, and failed to reduce the hernia. The pain being considerable, he had given the night before morphia subcutaneously, and changed the local applications from cold to hot.

I found the tumor very tense, and I withheld much manipulation, thinking it was useless to attempt reduction under the existing circumstances. We applied ice locally, and elevated the foot of the bed on a table. Later, at 8½ o'clock in the morning, when the parts were chilled by the ice, and hence less sensitive, taxis was tried, but it was evidently useless.

The idea had occurred to me before of making such a patient sneeze, while firm and well directed pressure on the hernia was being made, and I cast about to try the experiment; but no snuff could be found about the place. Finally, towards noon, some patent "catarrh snuff" was procured, which was used. While I had firm hold of the tumor, during the first good sneeze, I felt a little of the contents slip back into the abdominal cavity. The sneezing was kept up, and at times we could hear a little of the air in the protusion shoot through the internal ring. This happened at the end of the sneeze. I cannot explain it better than by saying there seems to be a billowy movement of the anterior wall of the abdominal cavity, from above downwards, which is suddenly reversed. This reversed action is accompanied by a sudden relaxation, as it were, at which instant a little of the contents of the hernial sac shoots back through the intestinal ring. The pressure was continued for over an hour—the omentum (for it was an entero-epiplocele), which undoubtedly caused the strangulation, being the last to disappear.

The other day, a gentleman was referred to me, at my office, who had a right oblique inguinal hernia, which I considered an epiplocele—the omentum only being strangulated. I failed at first in reduction by taxis, and left him in my office with ice on the tumor and his heels up in the air. After an hour, I returned with some Scotch snuff. I made him sneeze so long as there was any response in his Schneiderian membrane, meanwhile using diligent taxis. The tumor receded with more difficulty than I think an enterocele would under the same procedure, but finally it was all gone.

Whether the peculiar relaxation of the abdominal wall or the pulling within on the intestine or omentum during the sneeze does the good, or both combined, I cannot positively state, though I am inclined to the latter view. So far, however, I am very well pleased with my discovery, as I considered it; and I give it to the profession with the hope that, as a simple procedure, it may save many an unfortunate from the danger and suffering of a surgical operation.

P. S.—Since writing the above, I have been informed by Dr. S. D. Bowker, of Sunshine, Colorado, that Dr. Taylor, of Kansas City, suggested this snuff experiment to him some ten years ago. As the recent surgical authorities I have consulted do not mention it, and several physicians, including Prof. John T. Metcalfe, of New York, who was lately visiting our city, informed me they had never heard of it, I came to the conclusion that it was a new idea.

Dr. Bowker states that once while using taxis, in a case of direct inguinal hernia in a female, his patient sneezed, in consequence of the window being thrown open and a gust of air suddenly striking her, when the protusion as suddenly disappeared.—[Va. Med. Month.

